# Emergence of the cancer antigen PRAME as key player in epigenetic dysregulation and renal tumorigenesis

**DOI:** 10.1038/s41418-026-01700-1

**Published:** 2026-03-17

**Authors:** Paolo Salomoni

**Affiliations:** Nuclear Function Group, German Center for Neurodegenerative Diseases (DZNE), Bonn, Germany

**Keywords:** Oncogenes, Tumour biomarkers

The study by Zhang et al. [1] investigates the function of a nuclear cancer testis antigen, PReferentially expressed Antigen of MElanoma (PRAME) in renal cell carcinoma (RCC), which represents among the most aggressive human cancers and the most prevalent tumor of the urinary system. Furthermore, tumor-associated antigens are considered as promising targets, but often their functions in regulation of tissue homeostasis and cancer development remain only partially understood. Therefore, this study is timely and brings new understanding in a critically important area of cancer research.

PRAME is expressed in a variety of solid tumors as well as in myeloid malignancies, but it is hardly detected in normal tissues with the exception of testis and endometrium, making a broad cancer marker. Interestingly, in solid cancers, it is expressed in distant metastasis and predicts poor clinical outcomes. Instead, it is a favorable prognostic factor in myeloid tumors. The presence of PRAME peptides on major histocompatibility complex (MHC) has been suggested to potentially stimulate tumor-specific immunity. However, mechanisms controlling its expression and underlying its function in cancer remain only partially investigated. Notably, the authors show that PRAME expression is associated with distant metastases and poor prognosis also in clear cell renal cell carcinoma (ccRCC), confirming work on other solid tumors and providing further rationale for investigating its regulation and function in ccRCC. Loss- or gain-of-function experiments in RCC lines showed that PRAME can positively regulate tumor cell invasion. The effect was not limited to in-vitro cultures, as inhibition of PRAME in vivo results in inhibition of tumor growth as well as of metastasis to the lung. PRAME overexpression had the converse effect, ultimately indicating a pro-tumorigenic role of this cancer-associated marker.

The authors then turned their focus on investigating mechanisms controlling PRAME expression and implicate cooperation between DNA hypomethylation and C/EBPbeta in this context. This mechanism is demonstrated via comprehensive analysis of PRAME promoter and functional work based on pharmacological and genetic modulation of DNA methylation and C/EBPbeta. Importantly, C/EBPbeta expression is increased in ccRCC tissues and correlated with PRAME levels.

What would be the function of PRAME in ccRCC? The present study suggests that one of the main roles of PRAME is to repress the expression of NTN4 via engagement of the Polycomb Repressive Complex-2 (PRC2) as well as C/EBPbeta itself. This in turn would result in activation of AKT. Notably, PRAME role in transcriptional repression and functional interaction with PRC2 functional interaction has been demonstrated previously by others [2, 3] (further discussed below). The importance of the PRAME/PRC2/NTN4/AKT axis was demonstrated using pharmacological inhibitors complemented by elegant experiments using a peptide interfering with PRAME/EZH2 interaction. Finally, the clinical relevance of this pathway was assessed using paired paracancerous and ccRCC tissues, which showed that expression of PRAME, EZH2, phosphorylated AKT inversely correlated with NTN4 levels in tumors.

There are a number of questions arising from this interesting study. For instance, the genome-wide distribution of PRAME remains to be elucidated. For instance, it would be important to explore the association of PRAME with further PRC2 target genes and for instance, whether PRAME is found at bivalent genes or only monovalent, H3K27me3-enriched loci. Another area of potential investigation are tumors where gain of PRC2 function is a driver mechanism, such as in B-cell lymphomas [4]. With respect to renal cancer, a previous study linked high expression of EZH2 and CD8 + T-cell infiltration [5], which in my view is another aspect further investigating for what concerns the PRAME-centered axis described in the present study.

**Fig. 1:**
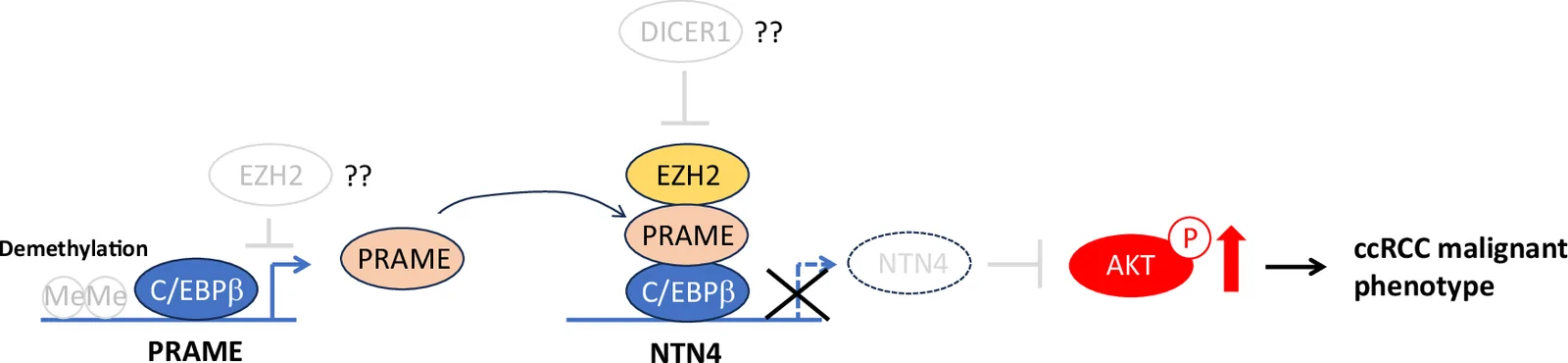
Working model showing that epigenetic regulation of both PRAME and NTN4 results in activation of AKT for induction of ccRCC malignant phenotype. In view of the reported EZH2-mediated repression of PRAME in lymphoma, it is possible that this level of regulation is not functional in ccRCC. Furthermore, the role of DICER1 in this axis remains to be elucidated, given that previous studies have reported increased synergy of PRAME/EZH2 in DICER Syndrome patients.

More generally, it would be interesting to understand why in the context of hematological malignancies PRAME expression is associated with better survival, even if also in APL and CML PRAME seems to work as transcriptional repressor for inhibiting myeloid differentiation and promoting transformation. One could speculate that its role in regulating proliferation and migration within the bone marrow leukemic niche could be relevant in controlling the undifferentiated state of leukemic cancer stem cells. Notably, PRAME is deleted in lymphomas, correlating with a cold tumor microenvironment and escape from cytoxic T-cell killing. Conversely, lymphomas carrying gain-of-function EZH2 mutations showed PRAME downregulation [6]. Finally, PRAME was shown to inhibit EZH2-mediated repressive function, providing a further level of complexity to the PRAME/EZH2 functional interactions (Fig. 1). This is another area for future investigation, as tumor-specific and context-specific effects may regulate the PRAME/EZH2 functional interaction across solid and hematological neoplasms.

Finally, PRAME and EZH2 interaction has been reported also in pituitary blastomas and other tumors arising in patients typified by the DICER1-related syndrome, which interestingly are associated with increased PI3K signaling [7, 8]. It would be important to investigate the PRAME/EZH2/NTN4/AKT axis also in the context of DICER-mutated neoplasms and see whether DICER loss-of-function alterations influence the functional interaction between PRAME and EZH2 and also if it may abrogate the inhibitory effects of EZH2 on PRAME expression observed in lymphomas (see above). Moreover, one could speculate that DICER1 alterations may occur also in ccRCC, as different types of kidney tumors arise in DICER1 syndrome patients [9]. More work is warranted in this exciting area of research.

Overall, the work by Zhang et al. provides novel insights into the function of a nuclear testis antigen, PRAME in renal carcinogenesis. This study may bear implications for other tumor entities, where PRAME has been implicated as tumor marker, but for which its function has not been elucidated. These findings highlight the complexity and context/tumor-specificity of epigenetic regulation and crosstalk with oncogenic signaling, serving as impetus for a better understanding of epigenetic (dys)regulation and plasticity in cancer.
